# The three-part model for coding causes and mechanisms of healthcare-related adverse events

**DOI:** 10.1186/s12911-022-01786-w

**Published:** 2022-02-24

**Authors:** Danielle A. Southern, James E. Harrison, Patrick S. Romano, Marie-Annick Le Pogam, Harold A. Pincus, William A. Ghali

**Affiliations:** 1 Centre for Health Informatics, Cumming School of Medicine, University of Calgary, Calgary, AB Canada; 2 College of Medicine and Public Health, Flinders University, Adelaide, Australia; 3 Division of General Medicine, University of California–Davis School of Medicine, Sacramento, CA USA; 4 Department of Epidemiology and Health Systems, Center for Primary Care and Public Health (Unisanté), University of Lausanne, Lausanne, Switzerland; 5 Department of Psychiatry, Columbia University and the New York State Psychiatric Institute, New York, NY USA; 6 Irving Institute for Clinical and Translational Research, Columbia University and New York-Presbyterian Hospital, New York, NY USA; 7 RAND Corporation, Pittsburgh, PA USA; 8 Office of Vice President of Research, University of Calgary, Calgary, AB Canada

**Keywords:** International Classification of Diseases, Adverse events, Quality and safety, ICD11

## Abstract

ICD-11 provides a promising new way to capture healthcare-related harm or injury. In this paper, we elaborate on the framework for describing healthcare-related events where there is a presumed causal link between an event and underlying healthcare-related factors. The three-part model for describing healthcare-related harm or injury in ICD-11 consists of (1) a healthcare-related activity that is the *cause* of injury or other harm (selected from Chapter 23 of ICD-11); (2) a *mode* or *mechanism* of injury or harm, related to the underlying cause (also from Chapter 23 of ICD-11); and (3) the harmful consequences of the event to the patient, selected from any of Chapters 1 through 22 of ICD-11 (most importantly, the injury or harm experienced by the patient). Concepts from these three elements are linked/clustered through *postcoordination* to reflect the three-part model in a single coded expression. ICD-11 contains many novel features, and the three-part model described here for healthcare-related adverse events is a notable example.

## Background

A patient suffers an intracranial hemorrhage related to an elevated INR caused by a drug interaction between warfarin (anticoagulation) and an antibiotic that they were taking for a urinary tract infection. (i.e. a rather complex clinical event).

Many healthcare quality and safety initiatives draw on International Classification of Diseases (ICD) coded data, including nationwide hospital outcome reports, hospital standardized mortality ratios, patient safety indicators, and risk adjustment indices [1]. Data coded to the 10th revision of ICD (ICD-10) have been used for some of these. ICD-10, however, is not optimal for capturing healthcare-related harms and injury events [2]. For example, ICD-10 does not allow users to link or associate multiple diagnosis concepts that relate to the same adverse event.

ICD-10 does have two features that can be used to describe multiple aspects of a case. First, for some types of events, a code from the 'Injury, poisoning and certain other consequences of external causes' chapter (Chapter 19) can be assigned, and a code from the 'External causes of morbidity and mortality' chapter (Chapter 20). Second, the dagger/asterisk system allows coding a symptom or manifestation of disease and its etiology. The existence of these ways to allow multiple ICD codes to better represent a case reflects a long-standing interest in the potential of doing so. However, implementation of the approach is incomplete in ICD-10, which lacks a systematic approach to link clinical factors with clinical consequences in a clear logic chain, e.g., when there may be a causal connection [3].

The 11th revision of ICD (ICD-11) provides a promising new way to capture healthcare-related harm or injury. Most crucially, ICD-11 allows the clustering or postcoordination of individual codes into code strings that create richer clinical descriptions by combining related clinical entities. Through the World Health Organization (WHO) ICD-11 revision process, there was a substantial restructuring of the healthcare-related injury content in ICD-10 chapters 19 and 20 to implement and adapt the postcoordination system for a rich and detailed description of healthcare-related adverse events. The Agency for Healthcare Research and Quality (AHRQ) Common Formats [4] and the WHO's former International Classification for Patient Safety (ICPS) [5] provided conceptual content that has informed ICD-11. The information model for describing adverse events in ICD-11 builds on the classification's embedded mechanism of postcoordination of coded concepts. It allows the coder to link the likely cause of an adverse event with the injury or other harm that resulted from it [2]. ICD-11 thus enables users to create very useful statements about what has happened to a patient. The approach to recording quality and safety events in ICD-11 is more flexible and systematic than in ICD-10, allowing more detailed case descriptions, yet it remains simple.

We now elaborate on and assess the 3-part model framework for describing healthcare-related events with a presumed causal link between an event and underlying healthcare-related factors. We then present some associated clinical examples that illustrate its use.

## Main text

### The three-part model

The 3-part model for capturing healthcare-related adverse events in ICD-11 consists of:

1. A healthcare-related activity that is the *cause* of injury or other harm (selected from Chapter 23 of ICD-11);
2. A *mode* or *mechanism* of injury or harm, related to the underlying cause (also from Chapter 23 of ICD-11); and
3. The harmful consequences of the event to the patient, selected from any of Chapters 1 through 22 of ICD-11 (most importantly, the injury or harm experienced by the patient).

Concepts from the three elements above are linked/clustered through *postcoordination* to reflect the three-part model in a single coded expression (examples to follow).

As shown in Table 1, the model divides causes of heath care-related harm or injury into four classes: 1. substances (drugs, medicaments and biological substances); 2. procedures (surgical and other); 3. devices (surgical and other devices, implants or grafts); and 4. other healthcare-related causes (e.g. problems associated with the physical transfer of patient, non-provision of necessary procedure, delayed diagnosis, fall in healthcare, etc.). As a cause of the adverse event, procedures, can be represented by any ICD-11 codes ranging from PK80 through PK8Z. Moreover, this approach permits the specification of various types of procedures (e.g. neurological procedures, cardiac procedures, etc.) and operative approaches to procedures (i.e., open vs. percutaneous vs. endoscopic approaches). Similarly, a device is represented by any code from PK90-PK9C, a code range that describes a wide variety of devices (e.g. pacemakers, other cardiac devices, catheters of various kinds, etc.) Finally, for substances as a cause, there is a single code to represent the cause (PL00—Drugs, medicaments or biological substances associated with injury or harm in therapeutic use), which can then be enriched in detail through postcoordination to extension codes (see codes presented in Chapter X of the ICD-11 Browser [6]) that specify the actual drug that caused harm (e.g. XM1MN4: heparin).

**Table 1:** Causes and associated modes from ICD-11 browser [6]

Causes of healthcare Related harm	Code for mode/mechanism	Code description
Surgical or other medical procedure	PL11	Mode of injury or harm associated with a surgical or other medical procedure
(any of codes PK80-PK8Z)	PL11.0	Cut, puncture or tear, as mode of injury or harm
	PL11.1	Burn arising during procedure, as mode of injury or harm
	PL11.2	Embolisation, as mode of injury or harm
	PL11.20	Air embolism, as mode of injury
	PL11.3	Foreign body accidentally left in body, as mode of injury or harm
	PL11.4	Failure of sterile precautions, as mode of injury or harm
	PL11.5	Procedure undertaken at wrong site or wrong side, as mode of injury or harm
	PL11.6	Pressure, as mode of injury or harm
Surgical or other medical device, implant or graft	PL12	Mode of injury or harm associated with a surgical or other medical device, implant or graft
(any of codes PK90-PK9C)	PL12.0	Structural device failure, as mode of injury or harm
	PL12.1	Functional device failure, as mode of injury or harm
	PL12.2	Perforation or protrusion by device, as mode of injury or harm
	PL12.3	Obstruction of device, as mode of injury or harm
	PL12.4	Dislodgement, misconnection or de-attachment, as mode of injury or harm
	PL12.5	Operator error, as mode of injury or harm
	PL12.6	Combination or interaction of operator error and device failure, as mode of injury or harm
Drug, medicament or biological substance	PL13	Mode of injury or harm associated with exposure to a drug, medicament or biological substance
(Code PL00)	PL13.0	Overdose of substance, as mode of injury or harm
	PL13.1	Underdosing, as mode of injury or harm
	PL13.2	Drug-related injury or harm in the context of correct administration or dosage, as mode of injury or harm
	PL13.3	Incorrect substance, as mode of injury or harm
	PL13.5	Incorrect administration of drug or medicament, as mode of injury
	PL13.50	Incorrect route of drug or medicament, as mode of injury
	PL13.51	Incorrect rate of drug or medicament, as mode of injury
	PL13.52	Incorrect timing of drug or medicament, as mode of injury
	PL13.53	Incorrect duration of drug or medicament, as mode of injury
	PL13.6	Medication or substance that is known to be an allergen, as mode of injury or harm
	PL13.7	Medication or substance that is known to be contraindicated for the patient, as mode of injury or harm
	PL13.8	Expired or deteriorated medication or substance, as mode of injury or harm
	PL13.9	Drug or substance interactions, as mode of injury or harm
	PL13.A	Inappropriate stoppage or discontinuation of drug, as mode of injury or harm
Other healthcare-related causes	PL14	Mode of injury or harm associated with other healthcare-related causes
(Code PL10)	PL14.0	Non-administration of necessary drug
	PL14.1	Non provision of necessary procedure
	PL14.2	Problem associated with physical transfer of patient
	PL14.3	Mismatched blood used in transfusion
	PL14.4	Other problem associated with transfusion
	PL14.5	Problem associated with physical restraints
	PL14.6	Problem associated with isolation protocol
	PL14.7	Problem associated with clinical documentation
	PL14.8	Problem associated with clinical software
	PL14.9	Incorrect diagnosis
	PL14.A	Delayed diagnosis
	PL14.B	Delayed treatment
	PL14.C	Patient received diagnostic test or treatment intended for another patient
	PL14.D	Problem associated with transitions of care, hand offs, or handovers
	PL14.E	Fall in healthcare

The manner in which an underlying cause actually produces injury or harm is represented by *a mode or mechanism* code selected from Chapter 23. The modes/mechanisms of harm are also presented in Table 1; they correspond to the four high-level categories of causes of healthcare-related harm described above but provide additional detail to characterize further how harm actually occurred. For example, when a medication causes an adverse event in a healthcare context, the new 3-part model permits the specification of precisely how harm occurred (i.e., an overdose of drug vs. drug interaction vs. incorrect drug vs. allergic reaction, etc.) Similarly, when a device causes harm, we can specify the mode or mechanism of harm (structural failure, perforation by a device, dislodgement of a device, etc.). For procedures, the mode/mechanism codes include concepts such as cut, embolization, foreign body accidentally left in the body, and failure of sterile precautions. Finally, when another healthcare-related factor causes harm, the mode/mechanism codes include concepts such as incorrect diagnosis, delayed treatment, fall in healthcare, transfusion reaction, etc.) Table 1 presents the complete listing of modes/mechanisms available in ICD-11—a substantial expansion of detail in this domain relative to ICD-10.

Recognizing that there are some instances where a healthcare-related adverse event has a clear high-level cause (e.g. “drug causing an event”), but an uncertain or unspecified mode/mechanism (i.e. overdose? Underdose? Interaction?), ICD-11 also provides an option to code *other specified and unspecified mode/mechanism* for each of the four categories of substances, procedures, devices, or other causes of healthcare-related harm.

A key element of the 3-part model is the ability to link and specify consequences (i.e., harm or injury) arising from a healthcare-related activity. These can be described in an entirely unconstrained manner by ICD-11 diagnosis codes residing anywhere in Chapters 1 through 22. Of relevance to healthcare quality and safety, there is a section of codes residing in Chapter 22 in a section entitled “Injury or harm arising from surgical or medical care, not elsewhere classified.” These specialized codes relate to a number of special healthcare-related injury conditions. However, the list is by no means comprehensive for describing healthcare-related harm or injury, and ICD-11 clustering allows coders to select any pertinent diagnostic codes from elsewhere in the classification.

### Clinical examples

Table 2 presents several examples of scenarios where healthcare-related harm has occurred.

**Table 2:** Examples for the ICD-11 quality and safety coding model

Causes of healthcare Related harm	Examples of harms^a^ and related causes and modes
Surgical or other medical procedure	*Example 1* A patient visits a primary care physician for removal of a skin lump, mainly to exclude the possibility of malignancy. The lesion is excised and the wound is sutured. It later becomes known that the physician had Hepatitis C and the patient has now contracted this disease *Harm* Acute hepatitis C 1E50.2 *Cause* Biopsy procedure, not elsewhere classified, associated with injury or harm in therapeutic use PK81.5 *Mode* Failure of sterile precautions, as mode of injury or harm PL11.4 Code Structure: 1E50.2/PK81.5/PL11.4 *Example 2* An patient is admitted due to a fractured neck of femur. Surgical fixation is undertaken. The operative site bleeds heavily the day after surgery, requiring return to theatre *Harm* Haemorrhage not elsewhere classified MG27 *Cause* Musculoskeletal procedure associated with injury or harm, open approach PK80.80 (Orthopaedic surgical procedures are included here) *Mode* Unspecified mode of injury or harm associated with a surgical or other medical procedure P11.Z (Note: Select PL11.Z because case documentation does not mention any specific mode or mechanism by which haemorrhage occurred) Code Structure: MG27/ PK80.80/PL11.Z
Surgical or other medical device, implant or graft	*Example 3* A patient had a left knee-replacement less than a year ago, because of arthritis. The implanted device has come loose, resulting in pain and reduced function *Harm* Pain in joint ME82; Specific Anatomy (use additional code, if desired) Knee joint XA8RL1; Laterality (use additional code, if desired)—Left XK8G *Cause* Orthopaedic devices associated with adverse incidents, prosthetic or other implants, materials or accessory devices PK99.2 *Mode* Dislodgement, misconnection or de-attachment, as mode of injury or harm PL12.4 Code Structure: ME82&XA8RL1&XK8G/PK99.2/PL12.4 *Example 4* Refractory urinary tract infection due to chronic indwelling catheter *Harm* Urinary tract infection, site and agent not specified GC08.Z *Cause* Gastroenterology or urology devices associated with adverse incidents, urinary catheter PK93.10 *Mode* Other specified mode of injury or harm associated with a surgical or other medical device, implant or graft PL12.Y (Note: Select PL12.Y because none of the more specific mode types appears to lead to infection of device) Code Structure: GC08.Z/PK93.10/PL12.Y
Drug, medicament or biological substance	*Example 5* A patient has been admitted to hospital for stabilisation of diabetes. They are erroneously prescribed three times the usual dose of an antidiabetic medication. The abnormally high dose is given, and the patient has a hypoglycaemic episode *Harm* Hypoglycaemia in the context of diabetes, unspecified 5A21 *Cause* Drugs, medicaments or biologic substances associated with injury or harm in therapeutic use PL00; Medication (use additional code, if desired)—Antidiabetic XM8S35 *Mode* Overdose of substance as mode of injury or harm PL13.0 Code Structure: 5A21/PL00&XM8S35/PL13.0 *Example 6* Patient presented to hospital with hallucinations due to malaria prophylaxis with mefloquine prescribed and taken at the correct dose *Harm* Visual hallucinations MB27.27 *Cause* Drugs, medicaments or biological substances associated with injury or harm in therapeutic use PL00; Medication (use additional code, if desired)—Mefloquine XM50J2 *Mode* Drug-related injury or harm in context of correct administration or dosage, as mode of injury or harm PL13.2 Code Structure: MB27.27/PL00&XM50J2/PL13.2
Other healthcare-related causes	*Example 7* Patient falls out of bed in a hospital and suffers a left hip fracture. The documentation describes that the nurse forgot to put the bedrails in place which lead to the patients fall *Harm* Fracture of neck of femur, unspecified NC72.2; Laterality (use additional code, if desired)—Left XK8G *Cause* Other healthcare-related causes of injury or harm PL10 *Mode* Fall in healthcare PL14.E Code Structure: NC72.2&XK8G/PL10/PL14.E *Example 8* Patient received an infusion of red blood cells and develops severe rigors that subside after an hour. It was discovered that there was a blood mismatch (not ABO or Rh incompatibility) *Harm* Other serum reactions NE80.3 *Cause* Other healthcare-related causes of injury or harm PL10 *Mode* Mismatched blood used in transfusion PL14.3 Code Structure: NE80.3/PL10/PL14.3

^a^Examples from WHO Reference Guide[7]

These examples are presented here, as they appear in the WHO ICD-11 Reference Guide [7] as relevant educational materials. For each example, the harm, cause and mode are described, along with a presentation of the postcoordinated coding string that should ultimately be coded in each case. There is no documented mode or mechanism in some instances (examples 2 and 4). In such cases, the additional category of 'mode unspecified' is available for coding to complete the 3-part model.

### Online resources to support coding of the 3-part model

**Fig. 1:**
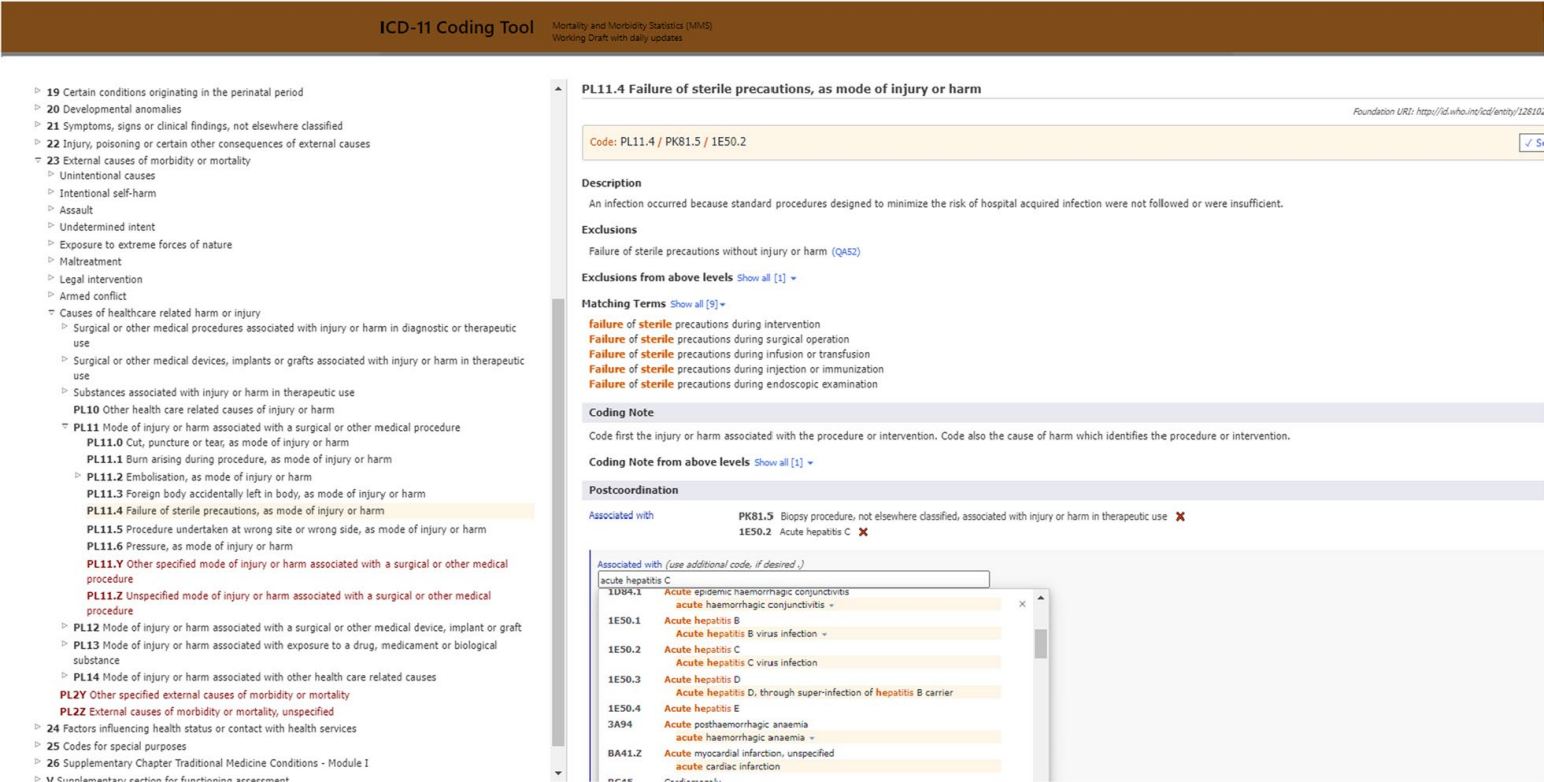
Screenshot of postcoordination in browser embedded in Coding Tool [8] for an example of Surgical or Other Medical Procedure

**Fig. 2:**
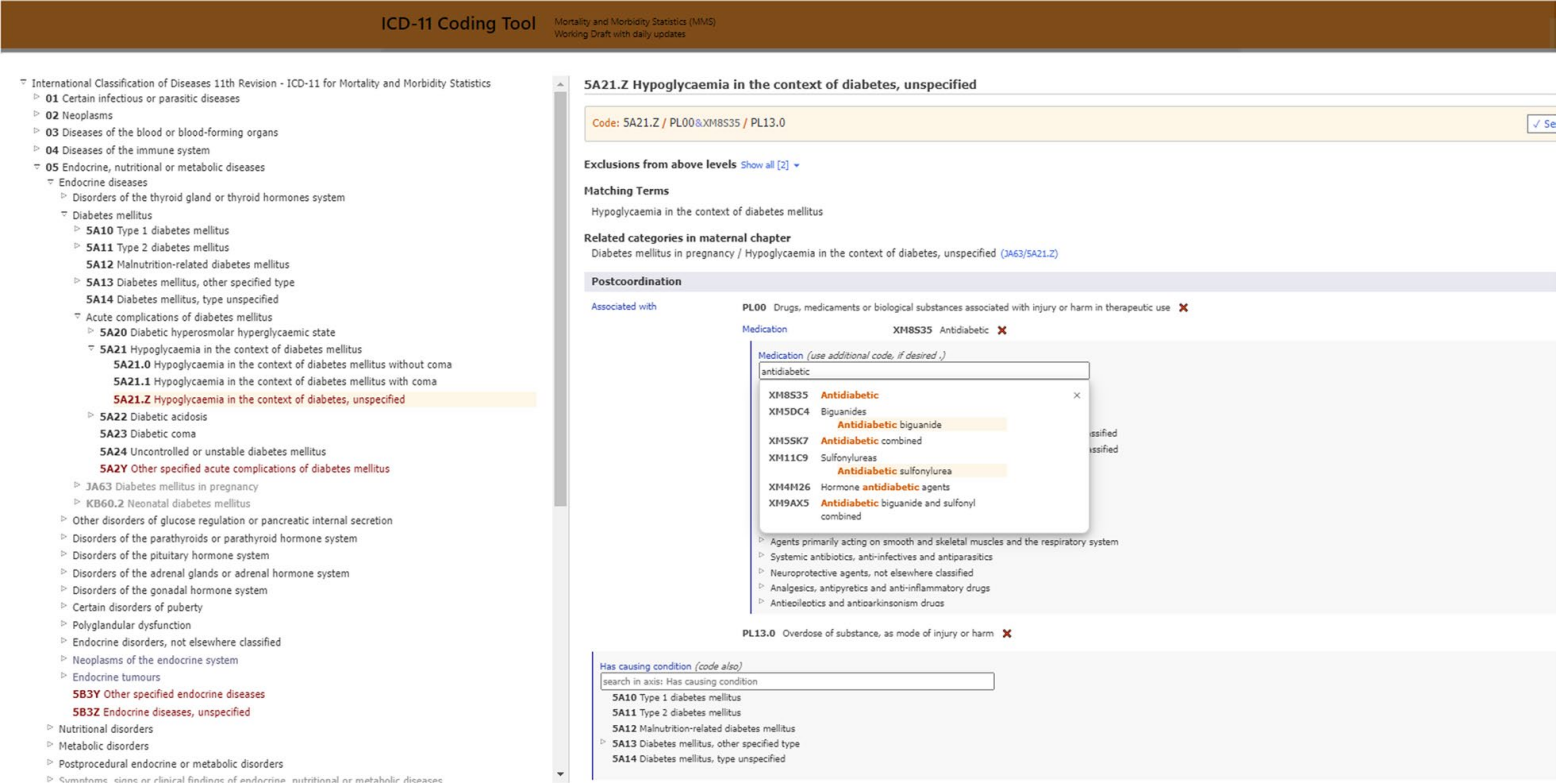
Screenshot of postcoordination in browser embedded in Coding Tool [8] for an example of Drug, Medicament or Biological Substance

One of the important advances in ICD-11 is the development of new online tools to assist coding of rich clinical data. Notably, the ICD-11 Browser [6] has an embedded open access Coding Tool [8] that allows dynamic detection of clinical concepts and the ability to go back and forth between the hierarchical Browser and a text-based search tool. Importantly, the WHO's online coding support tools provide postcoordination guidance, which is necessary given the added complexity associated with the 3-part model. Novice coders will need to receive training in the new 3-part model. However, even a trained and experienced coder is likely to benefit from being prompted by online tools to construct a 3-part code cluster. The online tool will support the production of better data coded more consistently across coders, facilities and nations. Figures 1 and 2 show screenshots of the tooling platform, demonstrating options to create a post-coordinated code. The two figures illustrate the postcoordination features relating to the examples presented in Table 2 (Fig. 1: a screenshot of tooling relating to Example 1, Fig. 2: Example 5).

## Conclusions

ICD-11 contains many new features, and its embedded postcoordination is of particular relevance as it provides a way to classify healthcare-related harms and injuries.

The 3-part model described here exemplifies how postcoordination unlocks the potential of ICD-11 to describe and codify complex clinical descriptions that sometimes arise in patient care. Field testing conducted during the ICD-11 development phase has assessed the utility of the proposed WHO framework for classifying patient safety events. Forster et al. found that the ICD-11 framework for healthcare-related adverse events has a high degree of coverage of relevant concepts [9]. With ICD-11, pilot testing on a wide scope of adverse event concepts has demonstrated comprehensive concept coverage, with most adverse events being readily codable [9].

However great the potential of ICD-11, transition to the new system will bring challenges, some of which are considered here. First, there is potential for the four high-level causes of healthcare-related harm to contribute to incidents where something undesirable has occurred, but without actually causing harm to a patient (e.g. incorrect medication administered to a patient without injury or harm; or a fall in a healthcare context without injury or harm). This is where a novel ICD-11 section in chapter 24 comes into play—i.e., ‘Healthcare-related circumstances influencing the episode of care without injury or harm.’ That relevant section of codes is discussed elsewhere in this manuscript series. Second, adverse events can occur in healthcare where the temporal relationship of healthcare contact in relation to the adverse event or new diagnosis is not entirely clear (e.g., pneumonia diagnosed in the hospital on the second day of a hospital stay or after surgery). This is where diagnosis timing extension codes become important—another promising ICD-11 coding feature discussed in another article in this manuscript series. Lastly, causation is a central question that is ubiquitously challenging to determine in all health information systems. An underlying prerequisite for the 3-part model to be used is that there is a clear causal link (documented in clinical records) between the causal factor and the injury/harm that are coded together in a cluster. However, there are many instances where causation is not always easy to infer from clinical documentation. When a relationship is non-causal, or when causation is unclear, the 3-part model should not be used—replaced instead by other coding approaches for describing healthcare-related events. These alternative approaches, and the specific situations where they apply, are described in a subsequent article in this manuscript series.

The increased richness of data and coding options in ICD-11 is not without challenges. For example, the 3-part adverse event framework described here has performed well in field testing conducted by a limited number of coders who had undergone training while also participating in developing new ICD-11 code content [10]. Now, however, the 3-part model needs to be tested in the hands of a global community of health information coders, using ICD-11 in a diversity of settings and situations.

Creating coding "guardrails" to prevent overuse, underuse, and misuse of the 3-part model in real-world postcoordination coding will be imperative for systems and training programs. Notably, the high-level question of when to use and when not to use, the 3-part framework is essential for coders to understand to capture such events adequately. Indeed, the tooling in ICD-11 (i.e., open access IT tools for coding assistance, developed by the WHO) discussed here is vital for aiding coders with the postcoordination. For example, both the Browser [6] and the coding tool [8] guide users in the postcoordination of the 3-part model. Lastly, cost implications for transitioning from an ICD-10 coded system to an ICD-11 coded system must also be considered.

Complex situations like the intracranial bleed caused by warfarin-antibiotic interaction frequently occur in healthcare settings [11]. However, current health information systems have a limited ability to capture and fully describe them through existing code sets.

ICD-11 has functionality that enables the capture of such events. In addition, the new classification's postcoordination and extension code mechanisms are key features that unlock the classification to produce a more dynamic and flexible health information system.

## Data Availability

Data sharing is not applicable to this article as no datasets were generated or analysed during the current study.

## Abbreviations

ICD: International Classification of Diseases
ICD-10: International Classification of Diseases, 10th revision
ICD-11: International Classification of Diseases, 11th revision
WHO: World Health Organization
AHRQ: The Agency for Healthcare Research and Quality
ICPS: International Classification for Patient Safety

## References

[1] Ghali WA, Pincus HA, Southern DA, Brien SE, Romano PS, Burnand B, Drösler SE, Sundararajan V, Moskal L, Forster AJ, Gurevich Y, Quan H, Colin C, Munier WB, Harrison J, Spaeth-Rublee B, Kastanjsek N, Üstün TB (2013). Icd-11 for quality and safety: overview of the who quality and safety topic advisory group. Int J Qual Healthcare.

[2] Makary M, Daniel M (2016). Medical error—the third leading cause of death in the US. BMJ.

[3] Southern DA, Pincus HA, Romano PS, Burnand B, Harrison J, Pickett D, Forster AJ, Moskal L, Quan H, Droesler SE, Sundararajan V, Collin C, Gurevich Y, Brien SE, Kostanjsek N, Üstün B, Ghali WA (2016). Enhanced capture of healthcare-related harms and injuries in the 11th revision of the International Classification of Diseases (ICD-11). Int J Qual Healthcare.

[4] Agency for Healthcare Research and Quality. Common Formats. https://www.pso.ahrq.gov/common. Accessed 25 Sept 2020.

[5] World Health Organization. The Conceptual Framework for the International Classification for Patient Safety (ICPS). https://www.who.int/patientsafety/implementation/taxonomy/ICPS-report/en/#:~:text=Overview-,The%20Conceptual%20Framework%20for%20the%20International%20Classification%20for%20Patient%20Safety,improving%20patient%20safety%20across%20systems. Accessed 25 Sept 2020.

[6] World Health Organization. ICD-11 Browser. https://icd.who.int/dev11/l-m/en. Accessed 15 July 2020.

[7] World Health Organization. ICD-11 Reference Guide. https://icd11files.blob.core.windows.net/refguide/html/index.html. Accessed 25 June 2020.

[8] World Health Organization. ICD-11 Coding Tool. https://icd.who.int/devct11/icd11_mms/en/current. Accessed 15 July 2020.

[9] Forster AJ, Burnand B, Droesler S, Gurevich Y, Harrison J, Januel JM, Romano PS, Southern DA, Sundararajan V, Quan H, Vanderloo SE, Pincus HA, Ghali WA (2017). A World Health Organization field trial assessing a proposed ICD-11 framework for classifying patient safety events. Int J Qual Healthcare.

[10] Eastwood C et al. Advancing data collection of hospital-related harms: validity of the new ICD-11 quality and safety use case. Int J Popul Data Sci. 2014; 3(4).

[11] Berry-Millett R, Bodenheimer TS (2009). Care management of patients with complex healthcare needs. Synth Proj Res Synth Rep.

